# Applying Dynamical Systems Theory to Improve Personalized Medicine Following Mild Traumatic Brain Injury

**DOI:** 10.1089/neur.2024.0040

**Published:** 2024-07-16

**Authors:** Shawn R. Eagle, Rebecca J. Henry

**Affiliations:** ^1^ Department of Neurological Surgery, University of Pittsburgh, Pittsburgh, Pennsylvania, USA.; ^2^ University College, Cork, Ireland.

**Keywords:** inflammasome, mild traumatic brain injury, mTBI, NLRP3, obesity

## Abstract

A sizable proportion of patients with mild traumatic brain injury (mTBI) have persistent symptoms and functional impairments months to years following injury. This phenomenon is continually observed despite an explosion of research and interest in improving mTBI clinical outcomes over the last two decades. All pharmacological clinical trials to date have failed to demonstrate improved outcomes for mTBI. One possible explanation for these continued failures is an overly myopic approach to treating mTBI (i.e., testing the effect of a single drug with a specific mechanism on a group of people with highly heterogenous injuries). Clinical presentation and prognosis of mTBI vary considerably between patients, and yet we continue to assess group-level effects of a homogenized treatment. We need to utilize an equally complex treatment approach to match the extraordinary complexity of the human brain. Dynamical systems theory has been used to describe systems composed of multiple subsystems who function somewhat independently but are ultimately interconnected. This theory was popularized in the motor control literature as an overarching framework for how the mind and body connect to interact and move through the environment. However, the human body can be viewed as a dynamical system composed of multiple subsystems (i.e., organ systems) who have isolated functions, which are also codependent on the health and performance of other interconnected organ systems. In this perspective piece, we will use the example of mTBI in the obese patient to demonstrate how broadening our approach to treatment of the individual (and not necessarily the injury) may ultimately yield improved outcomes. Furthermore, we will explore clinical and pre-clinical evidence demonstrating multiple system interactions in the context of obesity and TBI and discuss how expanding our understanding of the mechanistic interplay between multiple organ systems may ultimately provide a more personalized treatment approach for this mTBI patient subpopulation.

## Introduction

**FIG. 1.**
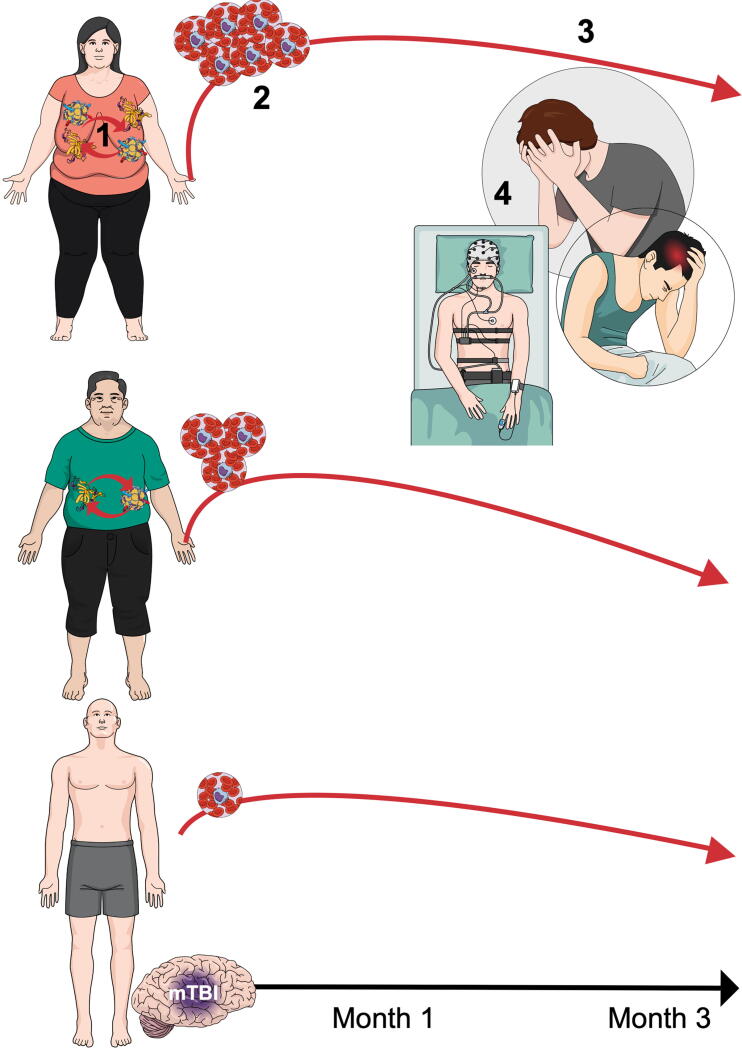
Schematic representation of our central hypothesis regarding adipose tissue-induced inflammation after mTBI. **1**). People with excess adipose tissue have higher baseline systemic inflammation. **2**). Following mTBI, a spike of inflammation is normal. We hypothesize that the patient with excess adipose tissue will have a higher inflammatory peak after mTBI. **3**). Inflammation will resolve at a slower pace for the patient with excess adipose tissue following mTBI. **4**). Excess and persistent inflammation increase odds for worse outcomes and known sequelae of both excess adipose tissue and mTBI (e.g., sleep apnea, depression, migraine, and so on).

Obesity is now considered a “global epidemic” with 13% of the global adult population considered obese.^1^ In addition, traumatic brain injury (TBI) is often described as a “silent epidemic” with an estimated 50 million individuals suffering each year;^2,3^ however, these numbers are likely a gross underestimation. Emerging evidence suggests that obese patients subsequently exposed to TBI suffer from worse outcomes, including prolonged symptomology^4^ and higher mortality rates,^5–8^ compared with patients with “normal” BMI. A majority of this evidence has been presented in the severe TBI population (i.e., those with a Glasgow Coma Scale [GCS] at presentation <8). However, based upon prior research and Centers of Disease Control estimates for obese BMI, approximately 1.1 million people in the United States have both functional limitations related to a “mild” TBI (mTBI; GCS = 13–15 at presentation) 1-year postinjury and obese BMI.^9^ There is high potential that obesity could mediate outcomes from mTBI, as well as severe TBI.^4,10,11^ Higher post-traumatic levels of circulatory obesity-induced inflammation, higher long-term levels of inflammation, and association with myriad comorbid conditions may be the leading mechanistic hypothesis for this relationship (see Fig. 1).^4,12^

### Overview of dynamical systems theory and application to human physiology

Dynamical systems theory (DST) became popularized in the 1980s as a means for understanding how we learn to interact with the world around us.^13^ Within the context of motor control and learning, DST describes accomplishing a given task as the interaction between multiple subsystems within the body, as well as interactions between our bodies and the environment within the constraints of the task itself.^14^ On a more comprehensive scale, the theory suggests that the human condition is usually in a steady state of “health” or “disease.” While perhaps appearing “steady” on the surface, either of these states is characterized by a complex interplay of feedforward and feedback mechanisms seeking to reinstate homeostasis after perturbations.^15,16^ Human existence shifts back and forth between health and disease steady states. The intensity of the shift itself can be gradual (as in the increased likelihood of disease states observed as we age) or abrupt.^16^ Abrupt shifts into a disease state can occur when one of the homeostatic mechanisms keeping us in a healthy state is rapidly disturbed by some stimulus.

A mechanical force resulting in mTBI is a clear example of an event which can rapidly shift our entire system into a disease state. The majority of research on understanding mTBI and how best to treat it have prioritized reductionist approaches aiming to treat the injury itself.^17,18^ Over the last 10–15 years, scientific and clinical communities treating patients with mTBI have broadened our understanding of mTBI recovery to include preexisting psychological, cognitive, and cortical-centric (e.g., headache/migraine, vestibular dysfunction) risk factors.^19–24^ Our knowledge of how to manage the postinjury sequelae of mTBI has also expanded, and it is now well-understood that mTBI can be “subtyped” for the purposes of targeting therapies to the individual’s specific needs.^25^ The purpose of this article is to present evidence that incorporating health of the bodily systems *below the cervical spine* into personalized management of patients with mTBI can improve outcomes and quality of life on a broader scale. DST can provide a viable framework by which to conceptualize and apply treatments for this heterogenous injury.

Appreciating the dynamic interaction of systems is better understood within the context of severe TBI, as systemic effects following severe TBI have been repeatedly noted in the liver, gastrointestinal, pulmonary, cardiovascular, kidney, and endocrine systems.^26–29^ Multisystem interactions are less well-characterized in the mTBI patient, possibly because serious or life-threatening changes to other organ systems are less likely to occur as a direct result of mTBI. The endocrine system is the primary exception, as multiple studies have identified endocrine system issues following mTBI.^30–32^ Izzy et al.^30^ recently conducted a study of 4,351 patients with mTBI and found increased risk for diabetes mellitus (hazard ratio = 1.9) and obesity (hazard ratio = 2.1). These studies have provided useful data for understanding how disturbing the central nervous system through mTBI can directly influence the downstream development of chronic issues in another organ system. In DST terminology, mTBI can act as an abrupt stimulus to transition the body into a disease system. However, we have very little information about how the *current* state of the system (at the time of injury) influences recovery from an mTBI.

**FIG. 2.**
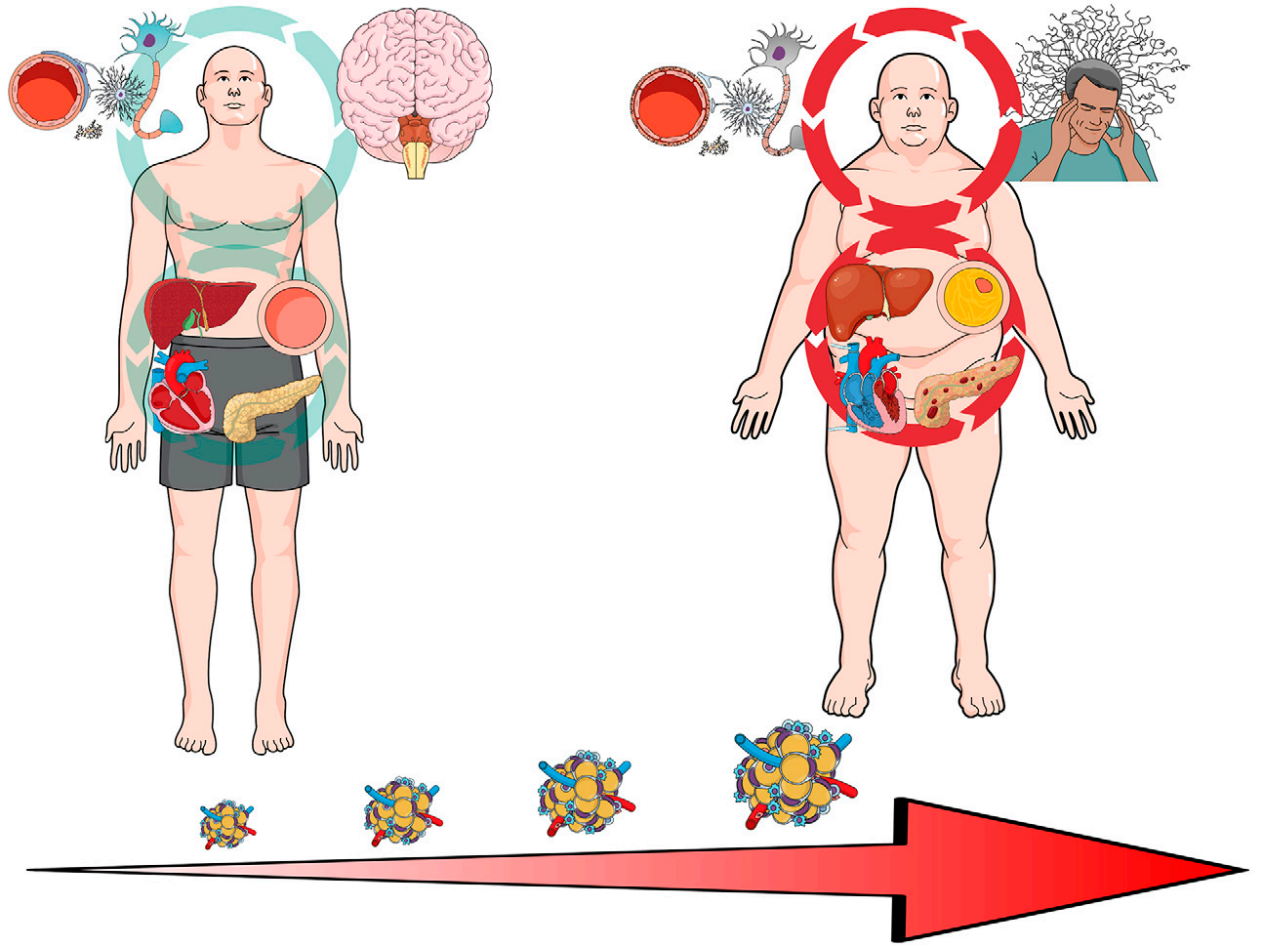
Depiction of a gradual shift from healthy bodily systems (left) with low circulatory inflammation, progressing over time to pathological interactions between bodily systems associated with obesity (right), increased circulatory inflammation, and impairment of microglia and cognitive function.

Obesity has been considered a steady disease state, but, in terms of DST, a more precise term may be “stable attractor”.^13^ As a person shifts into the obese disease state, a host of comorbidities and other medical conditions are “attracted” to the new conditions of the human body system (see Fig. 2). Multiple subsystems within the body dynamically adjust to excess calorie intake by storing adipose tissue within the capacity of that individual’s body.^33^ As this pattern continues, inflammatory cytokines and other cells increase in concentration within the blood.^33–35^ Blood lipids become more prevalent, and adipose tissue is deposited at higher than normal rates around the liver, viscera, kidneys, and pancreas ultimately leading to multiorgan system dysfunction and medical conditions such as insulin resistance and cardiovascular disease.^33^ Emerging evidence suggests that visceral fat itself should be viewed as an organ within the endocrine system, as it can self-generate hormone signals and inflammatory cells.^36^ Critically, inflammatory cells secreted by adipose tissue can cross the blood–brain barrier, increasing overall neuroinflammation and worsening clinical symptoms and cognitive performance.^37^ In other words, visceral fat is capable of creating a feedforward mechanism with the brain to maintain a stable disease state across multiple subsystems. The interaction of a physical trauma like mTBI into a stable disease system can theoretically exacerbate these feedforward processes into a deeper state of attraction.

### Interplay between obesity and traumatic brain injury in the pre-clinical setting

The pathophysiology of both obesity^38–40^ and TBI^41–44^ is characterized by widespread inflammation throughout the body, effects of which are associated with long-term neurological consequences.^39,44,45^ Experimentally, comorbid models of diet-induced obesity and TBI are associated with exacerbated neuroinflammatory responses and worsened neurological outcomes.^35,46–49^ Specifically, preexisting diet-induced obesity leads to an exacerbation of microglial activation in several brain regions, including the cerebral cortex, corpus callosum, and hypothalamus, at 30 days following exposure to mTBI.^47^ Notably, although exposure to mTBI in lean mice did not result in evident anxiety-like behavior, obese male mice subsequently exposed to mTBI exhibited increases in anxiety-like behavior.^47^ Further evidence demonstrating an interaction effect of obesity/diet and mTBI is that obese mice subsequently exposed to mTBI exhibited an exacerbation of central insulin resistance, microglial activation, and deficits in memory and anxiety-like behavior.^35^ Similarly, obese rodents subsequently exposed to moderate TBI displayed worsened short-term sensory and memory deficits^48^ and motor and learning/memory.^46^ Notably, such diet-induced exacerbations were associated with increases in expression of markers of neuroinflammation and oxidative stress and experimentally-induced lesion volume, up to one month postinjury.^46^

However, despite this, we still lack a clear understanding of the interplay between these two disease processes. Increasing our knowledge is not only important at a basic physiological level but also for the development of novel therapeutic targets for mTBI patients with a preexisting metabolic condition. Potential molecular pathways of interest from a therapeutic viewpoint include obesity-induced changes in gut microbiota, adiposity, and subsequent metabolic dysfunction.^50^ A recent study by *Henry* et al., demonstrated a potential bidirectional relationship between the adipose tissue and brain in a pre-clinical model of comorbid diet-induced obesity and moderate TBI.^49^ In this study, high-throughput transcriptomic analysis demonstrated a moderate TBI-obesity interaction of central and peripheral microglia/macrophage responses, including super-additive changes in several canonical inflammatory pathways. Notably, such interaction effects in cellular responses were associated with an obesity-induced exacerbation of cognitive deficits following exposure to moderate TBI. These findings are of potential importance as they show for the first time a bidirectional relationship between the adipose tissue and brain in the context of comorbid obesity and TBI, one that may be pharmacologically targetable.

In agreement with this, a previous body of research demonstrated a vital role for adipose tissue inflammation, specifically that of NOD-, LRR- and pyrin domain-containing protein 3 (NLRP3)-induced inflammation, in driving obesity-induced cognitive deficits; effects of which were shown to be mediated through IL-1-mediated microglial activation. Thus, suggesting that NLRP3/IL-1β signaling may underlie correlations between visceral adiposity and cognitive impairments in human obesity.^36^ Notably, findings from Henry et al. demonstrate that obesity-induced increases in visceral adipose tissue expression of NLRP3 and IL-1β were further exacerbated in the presence of moderate TBI. Thus, it is plausible to suggest that targeting of visceral adipose tissue in the context of comorbid obesity and mTBI may be a novel therapeutic target for patients with preexisting metabolic dysfunction, including obesity. However, it is important to note that these studies are focused on the impact of diet-induced obesity models on TBI outcomes; thus, perhaps not considering the complex nature of obesity etiology which may result from an array of factors, including genetics, stress, and other lifestyle factors. This will be an important consideration for future studies.

### Interplay between obesity and mTBI in the clinical setting

**FIG. 3.**
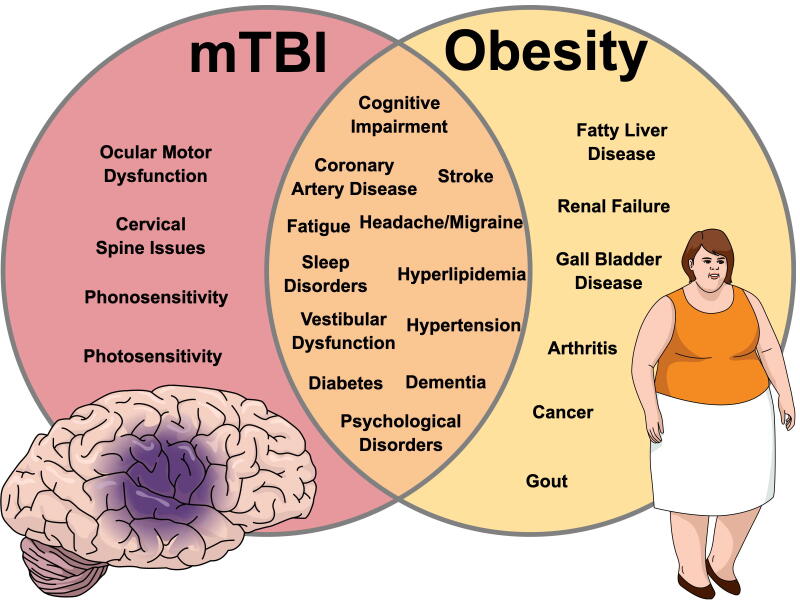
Venn diagram depiction of overlap between symptoms and medical conditions following mild traumatic brain injury (mTBI; left) and obesity (right).

One particularly interesting example of the multidimensional relationship between obesity as a disease state and mTBI can be found in the bariatric surgery literature. Recent case reports have studied severely obese patients who are suffering from chronic effects related to mTBI.^51^ Following bariatric surgery, the desired effect of weight loss and reversal of diabetes mellitus was achieved. Interestingly, there were also notable improvements in executive function and alleviation of symptoms the patient associated with their mTBI.^52^ These results are intuitive, as notable and sustained improvements in cognition, sleep, headache/migraine, and psychological health have been reported following bariatric surgery,^52^ which are domains with a high degree of overlap with commonly reported symptoms of mTBI (see Fig. 3). This evidence had led clinicians and researchers to suggest that bariatric surgery may be a useful treatment option for the severely obese patient experiencing chronic issues from mTBI. However, more conservative treatment options for managing mTBI in the obese patient should be considered first.

### Future directions for personalized mTBI treatments for patients with pre-existing metabolic dysfunction

#### Therapeutic potential for behavioral change and weight loss programs

Overwhelming evidence suggests that exercise and weight loss improve health and outcomes in the obese patient. A growing body of evidence also suggests that early aerobic exercise is beneficial to shortening recovery from mTBI. Early (following 24–48 h of relative rest) physical activity improves mTBI symptoms faster and reduces recovery time amongst adolescent athletes.^25^ A 2023 systematic review of 7 studies reported that prescribed physical activity reduces recovery time following sport-related concussion by 4.6 days.^53^ Amongst adolescents who adhered to a physical activity prescription following 72 h of relative rest, significant reductions in total symptoms were reported at 2-weeks postinjury in comparison to a control group, which waited to be asymptomatic at rest before initiating physical activity.^54^

Conversely, strict rest prolongs recovery time from mTBI and is associated with higher depression and anxiety symptoms.^55^ Varner et al.^56^ conducted the only known randomized controlled trial of early (>48 h of relative rest) physical activity as an intervention in adults with mTBI. The authors reported that a prescription of 30 min light exercise 5x per week for 1-month postinjury did not elicit a treatment effect compared with those with no instructions to exercise.^56^ Participants in the intervention group were instructed to perform light walking or stationary cycling “at a pace that does not cause you to sweat or breathe harder”.^56^ It is possible that this prescribed intensity was not strong enough to elicit a treatment effect, as more vigorous forms of aerobic exercise prescribed in both adult outpatients with persistent symptoms and adolescent athletes have consistently yielded positive results.^53,57^

Despite the intuitive connection, there is little evidence available that demonstrates the efficacy of exercise and/or weight loss interventions on improvements after mTBI in the obese patient. One small randomized controlled trial by Driver et al.^58^ in the moderate-severe TBI population showed that a prescribed weight-loss intervention significantly improved weight loss, blood pressure, triglycerides, and cholesterol in comparison to a control group. Results also suggested that the intervention group had better self-reported health habits than the control group within the first year. No data were reported from this study specific to TBI symptoms, such as cognition.

#### Pharmacological interventions for the obese patient with mTBI

A targeted anti-inflammatory drug for obese patients with mTBI is an obvious potential intervention for this population. Research has recently begun to investigate the impact of NLRP3-inhibitors on recovery from experimental brain injury in animal models. Ismael et al.^59^ reported that administration of MCC950, a NLRP3 inhibitor, significantly improved neurological function and reduced cerebral edema at 1 and 3 h post-TBI. MCC950 may inhibit inflammasome priming by downregulating nuclear factor kappa B and caspase-1, which are key components of inflammasome activation.^59^ Treatment with MCC950 also reduced NLRP3 expression and IL-1β release while improving short-term memory deficits in a blast exposure rat model.^60^ Neurodegenerative disease is associated with NLRP3 inflammation, and utilizing MCC950 has also been of interest in animal models to potentially reduce risk for chronic neurodegenerative disease. Naeem et al.^37^ recently demonstrated MCC950’s ability to reverse amyloid beta plaque formation and improve cognitive function in rats with Alzheimer’s Disease (AD). Future work should consider the potential role of NLRP3 inhibitors in this subpopulation of TBI patients of all severities.

#### Therapeutic potential of antiobesity drugs following mTBI

Other potential avenues of pharmacological intervention for this subpopulation of TBI patients of all severities may include antiobesity drugs. Glucagon-like peptide-1 (GLP) is a gastrointestinal peptide hormone that is secreted by the intestinal tract and mediates effects through the G-protein-coupled receptor (GLP1R).^61^ The long-acting GLP-1R agonist, semaglutide, has been approved by the FDA in both oral and injectable forms for the treatment of T2DM and in injectable forms for the treatment of obesity.^62^ In a double-blind Semaglutide Treatment Effect in People with Obesity (STEP) 1 trial, for 1961 nondiabetic adults with a BMI of 30 kg/m^2^, treated once weekly with semaglutide or placebo (2.4 mg; subcutaneous administration), for a total of 68 weeks, an average weight loss of 15.3 kg was reported in semaglutide treated participants.^63^ Importantly, both randomized STEP 3 and 8 clinical studies have reported similar effects on weight loss.^64,65^ The mechanisms of action of GLP-1R agonists on weight loss include a decrease in appetite and food intake, increased satiety, decreased gastric emptying and gastrointestinal motility, and a decrease in insulin resistance.^61^ However, there is now emerging evidence that GLP-1R agonists, in addition to effects on weight loss, can reduce inflammation in both mice and humans.^66–68^

Another potential antiobesity target that has received significant attention in recent years is the stress-regulated hormone, growth differentiation factor (GDF)-15, which is reported to decrease food intake, energy expenditure, and body weight in mice and nonhuman primates through the brainstem-restricted receptor, GDNF family receptor alpha-like.^69–71^ In a similar manner to that of GLP-1R agonists, GDF-15 is reported to exhibit anti-inflammatory effects in obese models.^72^ Given the evidence that targeting of systemic inflammatory responses is associated with beneficial effects on brain function in obesity,^36^ it is plausible to suggest that antiobesity drugs (GLP1R, GDF-15) may offer a potential novel avenue of investigation in the context of comorbid obesity and TBI of all severities, at least in part, using their ability to decrease inflammatory responses.

## Conclusion

Given the ever-increasing rates of obesity in modern society, it is plausible to suggest that more obese patients will suffer an mTBI throughout their lifetime. There is now increasing evidence that the presence of individual comorbidities, including but not limited to metabolic dysfunction, may play an important role in determining patients’ outcomes following exposure to mTBI. As such, it is important that we move away from the notion of a *‘magic bullet’* treatment for mTBI patients to that of a personalized treatment approach based on individual health factors throughout the person’s entire body. Viewing management of this injury through the lens of dynamic systems theory can ultimately lead to an improvement in patient outcomes and quality of life following mTBI

## Abbreviations Used

AD: Alzheimer’s Disease
BMI: Body Mass Index
DST: dDynamical Systems Theory
GCS: Glasgow Coma Scale
GDF: Growth Differentiation Factor
GLP: Glucagon-like Peptide-1
GLP1R: Glucagon-like Peptide 1 Receptor
IL: Interleukin
mTBI: mild traumatic brain injury
NLRP3: NOD-, LRR- and pyrin domain-containing protein 3
STEP: Semaglutide Treatment Effect in People with Obesity Trial
TBI: Traumatic Brain Injury

## References

[1] Chooi YC, Ding C, Magkos F. The epidemiology of obesity. Metabolism 2019;92:6–10; doi: 10.1016/j.metabol.2018.09.005 30253139

[2] Injury GBDTB, Spinal Cord Injury C. Global, regional, and national burden of traumatic brain injury and spinal cord injury, 1990-2016: A systematic analysis for the Global Burden of Disease Study 2016. Lancet Neurol 2019;18(1):56–87; doi: 10.1016/S1474-4422(18)30415-0 30497965 PMC6291456

[3] Dewan MC, Rattani A, Gupta S, et al. Estimating the global incidence of traumatic brain injury. J Neurosurg 2018;130(4):1080–1097; doi: 10.3171/2017.10.JNS17352 29701556

[4] Eagle SR, Puccio AM, Nelson LD, et al. Association of obesity with mild traumatic brain injury symptoms, inflammatory profile, quality of life and functional outcomes: A TRACK-TBI Study. J Neurol Neurosurg Psychiatry 2023;94(12):1012–1017; doi: 10.1136/jnnp-2023-331562 37369556

[5] Chabok SY, Yazdanshenas H, Naeeni AF, et al. The impact of body mass index on treatment outcomes among traumatic brain injury patients in intensive care units. Eur J Trauma Emerg Surg 2014;40(1):51–55; doi: 10.1007/s00068-013-0314-2 26815777

[6] Christmas AB, Reynolds J, Wilson AK, et al. Morbid obesity impacts mortality in blunt trauma. Am Surg 2007;73(11):1122–1125. 18092645

[7] Ditillo M, Pandit V, Rhee P, et al. Morbid obesity predisposes trauma patients to worse outcomes: A national trauma data bank analysis. J Trauma Acute Care Surg 2014;76(1):176–179; doi: 10.1097/TA.0b013e3182ab0d7c 24368375

[8] Mishra R, Galwankar S, Konar S, et al. Obesity as a predictor of outcome following traumatic brain injury: A systematic review and meta-analysis. Clin Neurol Neurosurg 2022;217:107260; doi: 10.1016/j.clineuro.2022.107260 35500420

[9] Nelson LD, Temkin NR, Dikmen S, et al. Recovery after mild traumatic brain injury in patients presenting to US level i trauma centers: A transforming research and clinical knowledge in traumatic brain injury (TRACK-TBI) study. JAMA Neurol 2019;76(9):1049–1059; doi: 10.1001/jamaneurol.2019.1313 31157856 PMC6547159

[10] Yue JK, Kobeissy FH, Jain S, et al. Neuroinflammatory biomarkers for traumatic brain injury diagnosis and prognosis: A TRACK-TBI Pilot Study. Neurotrauma Rep 2023;4(1):171–183; doi: 10.1089/neur.2022.0060 36974122 PMC10039275

[11] Huie JR, Diaz-Arrastia R, Yue JK, et al. Testing a multivariate proteomic panel for traumatic brain injury biomarker discovery: A TRACK-TBI Pilot Study. J Neurotrauma 2019;36(1):100–110; doi: 10.1089/neu.2017.5449 30084741 PMC6306686

[12] Yue JK, Cnossen MC, Winkler EA, et al. Pre-injury comorbidities are associated with functional impairment and post-concussive symptoms at 3-and 6-months after mild traumatic brain injury: A TRACK-TBI study. Front Neurol 2019;10:343. 31024436 10.3389/fneur.2019.00343 PMC6465546

[13] Turvey MT, Fonseca S. Nature of motor control: Perspectives and issues. Progress in Motor Control: A Multidisciplinary Perspective 2009:93–123. 10.1007/978-0-387-77064-2_6 PMC3726039 19227497

[14] Davids K, Glazier P, Araujo D, et al. Movement systems as dynamical systems: The functional role of variability and its implications for sports medicine. Sports Med 2003;33(4):245–260. 12688825 10.2165/00007256-200333040-00001

[15] Bigler ED. Systems biology, neuroimaging, neuropsychology, neuroconnectivity and traumatic brain injury. Front Syst Neurosci 2016;10:55. 27555810 10.3389/fnsys.2016.00055 PMC4977319

[16] Kenzie ES, Parks EL, Bigler ED, et al. Concussion as a multi-scale complex system: An interdisciplinary synthesis of current knowledge. Front Neurol 2017;8:513. 29033888 10.3389/fneur.2017.00513 PMC5626937

[17] Eagle SR, Collins MW, Dretsch MN, et al. Network analysis of research on mild traumatic brain injury in US military service members and veterans during the past decade (2010–2019). J Head Trauma Rehabil 2021;36(5):E345–E354; doi: 10.1097/HTR.0000000000000675 33741827

[18] Eagle SR, Kontos AP, Collins MW, et al. Network analysis of sport-related concussion research during the past decade (2010–2019). J Athl Train 2020; doi: 10.4085/280-20 PMC8063657 33543307

[19] Eagle SR.. Association of childhood psychological trauma with risk for positive dementia screening and depression in former professional football players-you injure the brain you have. JAMA Netw Open 2022;5(3):e223305; doi: 10.1001/jamanetworkopen.2022.3305 35315923

[20] Eagle SR, Brent D, Covassin T, et al. Exploration of race and ethnicity, sex, sport-related concussion, depression history, and suicide attempts in US youth. JAMA Netw Open 2022;5(7):e2219934; doi: 10.1001/jamanetworkopen.2022.19934 35796154 PMC9250048

[21] Eagle SR, Feder A, Manderino LM, et al. Concurrent validity of the Vestibular/Ocular Motor Screening (VOMS) tool with the Dizziness Handicap Inventory (DHI) among adolescents with vestibular symptoms/impairment following concussion. Phys Ther Sport 2022;53:34–39; doi: 10.1016/j.ptsp.2021.11.003 34785482

[22] Eagle SR, Kissinger-Knox AM, Feder A, et al. Temporal differences in concussion symptom factors in adolescents following sports-related concussion. J Pediatr 2022;245:89–94; doi: 10.1016/j.jpeds.2022.02.013 35157844

[23] Ferris LM, Kontos AP, Eagle SR, et al. Utility of VOMS, SCAT3, and ImPACT baseline evaluations for acute concussion identification in collegiate athletes: Findings from the NCAA-DoD Concussion Assessment, Research and Education (CARE) consortium. Am J Sports Med 2022;50(4):1106–1119; doi: 10.1177/03635465211072261 35179972

[24] Kontos AP, Monti K, Eagle SR, et al. False-positive rates and associated risk factors on the vestibular-ocular motor screening and modified balance error scoring system in US military personnel. J Athl Train 2022;57(5):458–463; doi: 10.4085/1062-6050-0094.21 35696602 PMC9205550

[25] Harmon KG, Clugston JR, Dec K, et al. American medical society for sports medicine position statement on concussion in sport. Br J Sports Med 2019;53(4):213–225. 30705232 10.1136/bjsports-2018-100338

[26] Krishnamoorthy V, Manley GT, Jain S, et al. Incidence and clinical impact of myocardial injury following traumatic brain injury: A Pilot TRACK-TBI Study. J Neurosurg Anesthesiol 2022;34(2):233–237; doi: 10.1097/ANA.0000000000000772 33901061 PMC8536798

[27] Volovici V, Steyerberg EW, Cnossen MC, et al. Evolution of evidence and guideline recommendations for the medical management of severe traumatic brain injury. J Neurotrauma 2019;36(22):3183–3189; doi: 10.1089/neu.2019.6474 31280663

[28] Snider SB, Temkin NR, Barber J, et al. Predicting functional dependency in patients with disorders of consciousness: A TBI-Model Systems and TRACK-TBI Study. Ann Neurol 2023;94(6):1008–1023; doi: 10.1002/ana.26741 37470289 PMC10799195

[29] Martinez-Perez R, Tsimpas A, Rayo N, et al. Role of the patient comorbidity in the recurrence of chronic subdural hematomas. Neurosurg Rev 2021;44(2):971–976; doi: 10.1007/s10143-020-01274-7 32146611

[30] Izzy S, Chen PM, Tahir Z, et al. Association of traumatic brain injury with the risk of developing chronic cardiovascular, endocrine, neurological, and psychiatric disorders. JAMA Netw Open 2022;5(4):e229478-e229478. 35482306 10.1001/jamanetworkopen.2022.9478 PMC9051987

[31] Claessen LÓE, Kristjánsdóttir H, Jónsdóttir MK, et al. Pituitary dysfunction following mild traumatic brain injury in female athletes. Endocr Connect 2024;13(2). 10.1530/EC-23-0363 PMC10831544 38078923

[32] Di Battista AP, Rhind SG, Churchill N, et al. Peripheral blood neuroendocrine hormones are associated with clinical indices of sport-related concussion. Sci Rep 2019;9(1):18605. 31819094 10.1038/s41598-019-54923-3 PMC6901546

[33] Afolabi HA, bin Zakariya Z, Shokri ABA, et al. The relationship between obesity and other medical comorbidities. Obesity Medicine 2020;17:100164.

[34] Delaney KZ, Vanstone CA, Weiler HA, et al. Regional adiposity and markers of inflammation in pre-school age children. Sci Rep 2018;8(1):15204; doi: 10.1038/s41598-018-33054-1 30315178 PMC6185945

[35] Karelina K, Sarac B, Freeman LM, et al. Traumatic brain injury and obesity induce persistent central insulin resistance. Eur J Neurosci 2016;43(8):1034–1043; doi: 10.1111/ejn.13194 26833850

[36] Guo D-H, Yamamoto M, Hernandez CM, et al. Visceral adipose NLRP3 impairs cognition in obesity via IL-1R1 on CX3CR1+ cells. J Clin Invest 2020;130(4):1961–1976. 31935195 10.1172/JCI126078 PMC7108893

[37] Naeem A, Prakash R, Kumari N, et al. MCC950 reduces autophagy and improves cognitive function by inhibiting NLRP3-dependent neuroinflammation in a rat model of Alzheimer’s disease. Brain Behav Immun 2024;116:70–84. 38040385 10.1016/j.bbi.2023.11.031

[38] Valdearcos M, Douglass JD, Robblee MM, et al. Microglial inflammatory signaling orchestrates the hypothalamic immune response to dietary excess and mediates obesity susceptibility. Cell Metab 2017;26(1):185–197 e3; doi: 10.1016/j.cmet.2017.05.015 28683286 PMC5569901

[39] Cope EC, LaMarca EA, Monari PK, et al. Microglia play an active role in obesity-associated cognitive decline. J Neurosci 2018;38(41):8889–8904; doi: 10.1523/JNEUROSCI.0789-18.2018 30201764 PMC6181311

[40] Bruce-Keller AJ, Keller JN, Morrison CD. Obesity and vulnerability of the CNS. Biochim Biophys Acta 2009;1792(5):395–400; doi: 10.1016/j.bbadis.2008.10.004 18992327 PMC2699212

[41] Faden AI, Barrett JP, Stoica BA, et al. Bidirectional brain-systemic interactions and outcomes after TBI. Trends Neurosci 2021;44(5):406–418; doi: 10.1016/j.tins.2020.12.004 33495023 PMC8084884

[42] Doran SJ, Henry RJ, Shirey KA, et al. Early or late bacterial lung infection increases mortality after traumatic brain injury in male mice and chronically impairs monocyte innate immune function. Crit Care Med 2020;48(5):e418–e428; doi: 10.1097/CCM.0000000000004273 32149839 PMC7541908

[43] Ritzel RM, Doran SJ, Barrett JP, et al. Chronic alterations in systemic immune function after traumatic brain injury. J Neurotrauma 2018;35(13):1419–1436; doi: 10.1089/neu.2017.5399 29421977 PMC5998829

[44] Henry RJ, Ritzel RM, Barrett JP, et al. Microglial depletion with CSF1R inhibitor during chronic phase of experimental traumatic brain injury reduces neurodegeneration and neurological deficits. J Neurosci 2020;40(14):2960–2974; doi: 10.1523/JNEUROSCI.2402-19.2020 32094203 PMC7117897

[45] Henn RE, Elzinga SE, Glass E, et al. Obesity-induced neuroinflammation and cognitive impairment in young adult versus middle-aged mice. Immun Ageing 2022;19(1):67; doi: 10.1186/s12979-022-00323-7 36550567 PMC9773607

[46] Ibeh S, Bakkar NZ, Ahmad F, et al. High fat diet exacerbates long-term metabolic, neuropathological, and behavioral derangements in an experimental mouse model of traumatic brain injury. Life Sci 2023;314:121316; doi: 10.1016/j.lfs.2022.121316 36565814

[47] Sherman M, Liu MM, Birnbaum S, et al. Adult obese mice suffer from chronic secondary brain injury after mild TBI. J Neuroinflammation 2016;13(1):171; doi: 10.1186/s12974-016-0641-4 27357503 PMC4928296

[48] Thomson S, Chan YL, Yi C, et al. Impact of high fat consumption on neurological functions after traumatic brain injury in rats. J Neurotrauma 2022;39(21–22):1547–1560; doi: 10.1089/neu.2022.0080 35658673

[49] Henry RJ, Barrett JP, Vaida M, et al. Interaction of high-fat diet and brain trauma alters adipose tissue macrophages and brain microglia associated with exacerbated cognitive dysfunction. bioRxiv 2023; doi: 10.1101/2023.07.28.550986 PMC11058055 38685031

[50] Shaito A, Hasan H, Habashy KJ, et al. Western diet aggravates neuronal insult in post-traumatic brain injury: Proposed pathways for interplay. EBioMedicine 2020;57:102829; doi: 10.1016/j.ebiom.2020.102829 32574954 PMC7317220

[51] McGlennon T, Buchwald J, Pories WJ, et al. Bypassing TBI: Metabolic surgery and the link between obesity and traumatic brain injury—A review. Obes Surg 2020;30(12):4704–4714. 33125676 10.1007/s11695-020-05065-3

[52] Dardano A, Aghakhanyan G, Moretto C, et al. Brain effect of bariatric surgery in people with obesity. Int J Obes 2022;46(9):1671–1677. 10.1038/s41366-022-01162-8 35729365

[53] Leddy JJ, Burma JS, Toomey CM, et al. Rest and exercise early after sport-related concussion: A systematic review and meta-analysis. Br J Sports Med 2023;57(12):762–770. 37316185 10.1136/bjsports-2022-106676

[54] Ledoux A-A, Barrowman N, Bijelić V, et al. PERC PedCARE Concussion team. Is early activity resumption after paediatric concussion safe and does it reduce symptom burden at 2 weeks post injury? The pediatric concussion assessment of rest and exertion (PedCARE) Multicentre Randomised Clinical Trial. Br J Sports Med 2022;56(5):271–278. 34836880 10.1136/bjsports-2021-105030

[55] Kontos AP, Eagle SR, Braithwaite R, et al. The effects of rest on concussion symptom resolution and recovery time: A meta-analytic review and subgroup analysis of 4329 patients. Am J Sports Med 2023;51(14):3893–3903; doi: 10.1177/03635465221150214 36847271

[56] Varner CE, Thompson C, de Wit K, et al. A randomized trial comparing prescribed light exercise to standard management for emergency department patients with acute mild traumatic brain injury. Academic Emergency Medicine 2021;28(5):493–501. 33481332 10.1111/acem.14215

[57] Alarie C, Gagnon I, Quilico E, et al. Physical activity interventions for individuals with a mild traumatic brain injury: A scoping review. Journal of Head Trauma Rehabilitation 2021;36(3):205–223. 33528174 10.1097/HTR.0000000000000639

[58] Driver S, McShan E, Swank C, et al. Efficacy of the diabetes prevention program group lifestyle balance program modified for individuals with TBI (GLB-TBI): results from a 12-month Randomized Controlled Trial. Annals of Behavioral Medicine 2023;57(2):131–145. 35775789 10.1093/abm/kaac036

[59] Ismael S, Nasoohi S, Ishrat T. MCC950, the selective inhibitor of nucleotide oligomerization domain-like receptor protein-3 inflammasome, protects mice against traumatic brain injury. J Neurotrauma 2018;35(11):1294–1303. 29295651 10.1089/neu.2017.5344 PMC5962912

[60] Ravula AR.. Repeated Low-Level Blast Induces Chronic Neuroinflammation and Neurobehavioral Changes in Rat Models. New Jersey Institute of Technology; 2022.

[61] Wang JY, Wang QW, Yang XY, et al. GLP-1 receptor agonists for the treatment of obesity: Role as a promising approach. Front Endocrinol (Lausanne);2023;14:1085799; doi: 10.3389/fendo.2023.1085799 36843578 PMC9945324

[62] Cowart K.. Oral semaglutide: First-in-class oral GLP-1 receptor agonist for the treatment of type 2 diabetes mellitus. Ann Pharmacother 2020;54(5):478–485; doi: 10.1177/1060028019889064 31744308

[63] Wilding JPH, Batterham RL, Calanna S, et al. Once-weekly semaglutide in adults with overweight or obesity. N Engl J Med 2021;384(11):989–1002; doi: 10.1056/NEJMoa2032183 33567185

[64] Rubino DM, Greenway FL, Khalid U, et al. Effect of weekly subcutaneous semaglutide vs daily liraglutide on body weight in adults with overweight or obesity without diabetes: The step 8 Randomized Clinical Trial. JAMA 2022;327(2):138–150; doi: 10.1001/jama.2021.23619 35015037 PMC8753508

[65] Wadden TA, Bailey TS, Billings LK, et al. Effect of subcutaneous semaglutide vs placebo as an adjunct to intensive behavioral therapy on body weight in adults with overweight or obesity: The STEP 3 Randomized Clinical Trial. JAMA 2021;325(14):1403–1413; doi: 10.1001/jama.2021.1831 33625476 PMC7905697

[66] Eguchi Y, Kitajima Y, Hyogo H, et al. Pilot study of liraglutide effects in non-alcoholic steatohepatitis and non-alcoholic fatty liver disease with glucose intolerance in Japanese patients (LEAN-J). Hepatol Res 2015;45(3):269–278; doi: 10.1111/hepr.12351 24796231

[67] Lee YS, Park MS, Choung JS, et al. Glucagon-like peptide-1 inhibits adipose tissue macrophage infiltration and inflammation in an obese mouse model of diabetes. Diabetologia 2012;55(9):2456–2468; doi: 10.1007/s00125-012-2592-3 22722451

[68] Somm E, Montandon SA, Loizides-Mangold U, et al. The GLP-1R agonist liraglutide limits hepatic lipotoxicity and inflammatory response in mice fed a methionine-choline deficient diet. Transl Res 2021;227:75–88; doi: 10.1016/j.trsl.2020.07.008 32711187

[69] Hsu JY, Crawley S, Chen M, et al. Non-homeostatic body weight regulation through a brainstem-restricted receptor for GDF15. Nature 2017;550(7675):255–259; doi: 10.1038/nature24042 28953886

[70] Mullican SE, Lin-Schmidt X, Chin CN, et al. GFRAL is the receptor for GDF15 and the ligand promotes weight loss in mice and nonhuman primates. Nat Med 2017;23(10):1150–1157; doi: 10.1038/nm.4392 28846097

[71] Yang L, Chang CC, Sun Z, et al. GFRAL is the receptor for GDF15 and is required for the anti-obesity effects of the ligand. Nat Med 2017;23(10):1158–1166; doi: 10.1038/nm.4394 28846099

[72] Tsai VW, Zhang HP, Manandhar R, et al. Treatment with the TGF-b superfamily cytokine MIC-1/GDF15 reduces the adiposity and corrects the metabolic dysfunction of mice with diet-induced obesity. Int J Obes (Lond) 2018;42(3):561–571; doi: 10.1038/ijo.2017.258 29026214

